# Physicians’ Perspectives on HL7 Information Policy Sensitive Value Set: A Validation Study through Health Concept Categorization

**DOI:** 10.3390/healthcare11212845

**Published:** 2023-10-28

**Authors:** Maheswari Eluru, Daniel Hector Mendoza, Audrey Wong, Mohammad Jafari, Michael Todd, Patricia Bayless, Darwyn Chern, Christina Eldredge, Rodrigo Fonseca, Pedro Franco-Fuquen, Juan Esteban Garcia-Robledo, Benjamin Grant Gifford, Rhea Hans, Eider Felipe Moreno-Cortes, Ajay Perumbeti, Fabio Samir Vargas-Cely, Lin Zhao, Maria Adela Grando

**Affiliations:** 1 College of Health Solutions, Arizona State University, Phoenix, AZ 85054, USAdhmendoz@asu.edu (D.H.M.);; 2 Health Level Seven International, Ann Arbor, MI 48104, USA; 3 Edson College of Nursing and Health Innovation, Arizona State University, Phoenix, AZ 85004, USA; mike.todd@asu.edu; 4 District Medical Group, Phoenix, AZ 85016, USA; 5 Copa Health, Phoenix, AZ 85009, USA; darwyn.chern@copahealth.org; 6 Morsani College of Medicine, University of South Florida, Tampa, FL 33602, USA; 7 Mayo Clinic, Phoenix, AZ 85054, USA; fonseca.rodrigo@mayo.edu (R.F.); vargascely.fabio@mayo.edu (F.S.V.-C.); 8 HonorHealth, Phoenix, AZ 85020, USA; 9 College of Medicine, University of Arizona, Phoenix, AZ 85004, USA; 10 Banner Health Systems, Phoenix, AZ 85006, USA

**Keywords:** data protection and privacy, HL7 terminology, granular patient consent, information sensitive data sharing

## Abstract

The Health Level 7 (HL7) organization introduced the Information Sensitivity Policy Value Set with 45 sensitive data categories to facilitate the implementation of granular electronic consent technology. The goal is to allow patients to have control over the sharing of their sensitive medical records. This study represents the first attempt to explore physicians’ viewpoints on these categories. Twelve physicians participated in a survey, leading to revisions in 21 HL7 categories. They later classified 600 clinical data items through a second survey using the updated categories. Participants’ perspectives were documented, and data analysis included descriptive measures and heat maps. In the first survey, six participants suggested adding 19 new categories (e.g., personality disorder), and modifying 25 category definitions. Two new categories and sixteen revised category definitions were incorporated to support more patient-friendly content and inclusive language. Fifteen new category recommendations were addressed through a revision of category definitions (e.g., personality disorder described as a behavioral health condition). In the second survey, data categorizations led to recommendations for more categories from ten participants. Future revisions of the HL7 categories should incorporate physicians’ viewpoints, validate the categories using patient data or/and include patients’ perspectives, and develop patient-centric category specifications.

## 1. Introduction

Patients want granular control over the sharing of their medical records [1]. The Office of the National Coordinator (ONC) for Health Information Technology defined granular choice as “a detailed choice an individual makes to share specific types of health data” [2]. The ONC envisions that granular privacy consent directives will enable the capture and exchange of patients’ preferences to advance care coordination in multiple settings for treatment, payment, healthcare operations, and research. Granular privacy consent improves patient user accessibility and engagement, allows for preference-based customization, and increases data security. However, challenges include age and digital literacy concerns. [3]

In the context of health data, sensitive data categories refer to those types of information that are considered highly sensitive or private, and therefore require extra precautions to ensure their confidentiality. Patients have reported fears of stigma and a desire to restrict access to sensitive data, including those related to behavioral health [3], demographics [4], diagnoses [5], disabilities [6], drugs [3], genetic diseases [3], infectious diseases [7], sexual and reproductive health [3], social determinants of health [7], and violence [8].

Categorizing sensitive health data is an important step in ensuring that the data is protected for several reasons. First, it ensures that the appropriate security measures are in place to protect this information from being accessed or misused by unauthorized people [3]. Second, it can assist organizations and healthcare professionals in adhering to the laws and rules that control gathering, storing, and using sensitive health information [3]. Healthcare organizations and professionals must navigate compliance with federal and state laws (for instance, 42 U.S.C. § 264 for communicable diseases) regarding the privacy of personal health information. Ensuring that patients are fully aware of their rights and the legal consequences of disclosing or withholding their personal health information requires transparent communication and education.

To support the development of consent-based granular data sharing technology that supports patient choices and state and federal legal requirements, the Substance Abuse and Mental Health Services Administration (SAMHSA) [8], the National Committee on Vital and Health Statistics (NCVHS) [9] and the Health Level Seven (HL7) [10] organization have proposed data sensitivity categories.

SAMHSA proposed the following sensitive data categories: alcohol use and alcoholism, drug use, genetic data, HIV/AIDS, mental health, sexual and reproductive health, other addictions, and other communicable diseases. In addition, SAMHSA developed Consent2Share, an open-source consent technology to support granular consent options aligned with federal and state data-sharing requirements [11]. The software was pilot tested using 1080 medical record items extracted from the EHRs of 36 patients with behavioral health conditions [9]. When the tool and health providers classified medical record items using the SAMHSA sensitive data categories, significant differences were found (χ^2^ (2, N = 584) = 114.74, *p* ≤ 0.0001). Sensitivity comparisons led to 56.0% agreements, 31.2% disagreements, and 12.8% partial agreements. Also, Consent2Share was pilot tested with 199 English- and Spanish-speaking patients with behavioral health conditions and patient guardians [8]. All participants desired granular control over the sharing of their health data. A majority (83%) indicated that the supported sensitive data categories satisfied their data-sharing privacy preferences.

The NCVHS has identified five sensitive data categories that require special handling to protect patient privacy needs, including mental health, sexual and reproductive health, domestic violence, substance abuse, and genetic information [12]. The NCVHS is aware that sensitive data views vary among people. The NCVHS acknowledges that classifying sensitive material into specific categories and specifying which pieces of information fall under each category “will be a complex and demanding job”. Despite this, they consider defining specific types of sensitive health information crucial.

HL7 is a non-profit organization that creates standards for electronic health information exchange, integration, sharing, and information extraction [13]. The HL7 standards [14], including the Fast Healthcare Interoperability Resources (FHIR), are widely used in the healthcare industry to facilitate information exchange between various healthcare systems and applications [15]. HL7 has put forward an HL7 terminology version, 5.1.0, that includes Information Sensitivity Policy Value Sets comprising 45 sensitive data categories [16].

To our knowledge, physicians or patients have not validated the HL7 Information Sensitivity Policy categories. Therefore, we aim to assess physician perspectives on the HL7 Information Sensitivity Policy categories and their potential to support granular electronic medical record sharing.

## 2. Methods

### 2.1. FHIR Patient Synthetic Data Access

In total, 2780 data items were extracted from 26 synthetic patient medical records codified in the FHIR standard [17]. The data items corresponded to medications, laboratory tests, diagnoses, demographic data, allergies, and procedures/services. A total of 2072 of the 2780 data items were duplicates (present in more than one patient synthetic data). The resulting 708 non-duplicated data items were randomly divided into six sets of approximately 100 data items each (Supplementary S1).

### 2.2. Physician Recruitment

The Institutional Review Board (IRB) at Arizona State University approved the study #STUDY00017492 on 1 March 2023, which asked physicians for written consent to participate in two electronic surveys.

Age ≥ 18 years, English-speaking physicians with an MD, DO, or MBBS degree were the inclusion criteria for participation in the study. To initiate the recruitment process, a designated member of our research team proactively contacted a group of physicians known to us through professional networks. These initial contacts served as a starting point for the word-of-mouth recruitment approach, with the recruited physicians subsequently referring their colleagues who met the inclusion criteria. This snowballing recruitment method allowed us to access a diverse group of qualified participants. Participants who completed two surveys were invited to help revise the manuscript and approve the final version.

### 2.3. Sensitive Data Categories

Forty-five HL7 sensitive data categories were combined with seven additional sensitive data categories proposed by Banerjee et al. (i.e., danger to others and themselves, disabilities, infectious diseases, pain management, social determinants of health, sexual health, and sexually transmitted diseases). Fifteen duplicate categories were removed, and the resulting 10 categories (parent) and 27 subcategories (children) are presented in Table 1. All sensitive data categories were provided with definitions from reputable sources (e.g., WHO) (Supplementary S2).

### 2.4. First Survey

The aim was to recruit 12 physicians for the initial online survey, which was developed to acquire physician feedback on the proposed sensitive data taxonomy (Supplementary S3).

After participants gave consent to participate in the study and completed five demographic questions, instructions directed them to provide feedback on the proposed sensitive data categories (Table 1) and their definitions. This was followed by opportunities to provide general feedback on the sensitive data categories (i.e., proposed categories were sufficient, fewer categories were required, and more categories were required than proposed).

The survey responses and physician feedback were used to modify the proposed sensitive data categories into 10 categories (parent) and 23 subcategories (children) and their definitions. The second survey employed the revised artifacts for categorizing health data items.

### 2.5. Second Survey

All 12 participants finished the initial survey and agreed to participate in a second online survey (Supplementary S3). The participants were then divided into six pairs. For the second survey, a total of 600 data items were selected for participants to categorize into the revised sensitive data categories developed using the feedback collected from the initial survey. Each pair of participants was randomly assigned 100 of the 600 data items to categorize. Participants could categorize each health data item into one or more categories or choose “other” if they were unsure about a health data item or believed that it did not fit into one of the proposed categories. Information button links provided definitions for sensitive data categories. As with the initial survey, participants were also asked to provide feedback regarding the overall sufficiency of the resulting sensitive data categories.

### 2.6. Data Analysis

For the first survey, participants’ suggestions related to (a) renaming, (b) removing or relocating, and (c) redefining each category were tallied (1 point per suggestion) for each of the ten initial categories given in the first column of Table 1. The tabular summary of these counts was augmented by applying a heat map color scheme to help to visualize the differential frequency of suggestions across combinations of categories and suggestion types.

For the second survey, within each pair, sensitive data categorizations were classified as agreeing, partially agreeing, or disagreeing. Agreement occurred when both participants in a pair assigned the exact same categories to an item. For example, both participants in a pair classified a social security number as demographic information. Partial agreement occurred when both participants in a pair assigned an item such that it had at least one category in common across raters within the pair. For example, one participant classified marital status as demographics, while another classified it as demographics and social determinants of health. There was disagreement when two participants’ assignments for an item had no category in common. For example, if one participant classified the lab test corresponding to throat culture as a diagnosis, and the second participant classified it as “Other”. The number of agreements, partial agreements, and disagreements was computed for each of the 11 categories in the second survey and visualized using a heat map.

We then assessed the relative instability of each category and compared them across the first and second surveys. Using data from the first survey, we computed the number of suggested changes across all categories (total sum of suggested changes) and the number of suggested changes within each category (category sum of suggested). Suggestions to classify categories as “Other” were not counted toward either the total sum or a category sum, as this category was not part of the initial proposed taxonomy. Instability for each category in the first survey was operationalized as the category sum divided by the total sum (a proportion). Using the data from the second survey, we then computed a weighted sum of within-pair partial agreements (each weighted 0.5) and disagreements (each weighted 1.0) across all 600 data items (total sum) and separately for the data items within each category (category sum). Instability within each category in the second survey was operationalized as the category sum divided by the total sum. These category-specific instability values were then compared across surveys and visualized using a heat map.

## 3. Results

### 3.1. Demographics

Table 2 presents a comprehensive overview of the study demographics, collected through the first survey. The results indicate that most participants were over 30 (58.34%), white (41.66%), male (66.67%), and had graduated from medical school within the past five years (58.33%). Moreover, in line with the study’s design, the participants represented distinct medical specialties.

### 3.2. Perceptions on HL7 Information Sensitive Data Categories

#### 3.2.1. First Survey

The first survey served as a preliminary validity check for the HL7 Information Sensitivity Policy categories and definitions (Table 3) by gathering feedback and comments from practicing physicians. Participants made 20 suggestions for adding 19 new categories, of which 2 were supported by the literature and incorporated into the second survey (see Table 4 (1)). Participants also provided a total of 74 suggestions for relocating (7 suggestions), renaming or removing (5 suggestions), and redefining (62 suggestions) categories. We implemented changes corresponding to a handful of these other suggestions by relocating 2 categories (Table 4 (2)), renaming or removing categories (Table 4 (3)), and redefining 16 categories (Table 4 (4)).

In the initial taxonomy, “Diagnoses”, which has no subcategories (least number), emerged as the most stable category, with only one request for definition revision (Table 3). On the other hand, behavioral health, with its eight subcategories (higher number), exhibited the least stability, garnering 19 suggestions for improvement (1 suggestion to relocate a category, 3 suggestions to remove or rename a category, and 15 suggestions to change category definitions).

In the initial survey, the participants recommended enhancing the patient friendliness and inclusiveness of the HL7 Information Sensitivity Policy categories’ names and definitions. Patient-friendly language focuses on the patient’s well-being and places them as the central focus of care [18]. It underscores the importance of establishing an inclusive environment that embraces diversity, advocates for equality, and encourages the active involvement of all individuals. Of the 94 total comments received for adding, renaming, or removing, relocating, and redefining sensitive data categories, 21 (22.34%) were recommendations for more inclusive and patient-friendly vocabulary. For instance, “*… I agree women experience assault more. But the way it’s written makes it seem like men do not experience assault*” recommended higher inclusiveness in the “Sexual assault, abuse or domestic abuse” definition (see Table 4 (4)).

The results of the first survey revealed divided opinions regarding the sufficiency of the sensitive data categories. Five (41.66%) of the twelve participants indicated that the proposed categories adequately captured data sensitivity, and seven (58.33%) expressed a need for additional categories. For instance, they recommended adding the categories “Healthy diet” and “Psychiatry”. One participant initially agreed that the number of categories was sufficient (Table 1). However, after reviewing the definitions of the categories (Supplementary S4), that participant requested additional categories.

#### 3.2.2. Second Survey

An examination of the responses to the second survey revealed that across all 600 health data items categorized by the six pairs of participants, there were 219 (36.50%) agreements, 149 (24.83%) partial agreements, and 232 (38.67%) disagreements. The “Medications” category had the highest number of agreements, representing 37 out of 219 (37.76%) of the total agreements, and the category with the highest number of disagreements was “Diagnoses”, accounting for 126 out of 232 (53.16%) of the total disagreements (Table 5). Also notable was the frequent utilization of the “Other” category by participants during the categorization process, which was selected in 136 out of 600 instances (22.66%).

The second survey revealed that the majority (83.33%) of the participants expressed the need for additional categories to be included. Only one participant (8.33%) agreed that the proposed categories adequately captured the sensitivity of the data, while another participant (8.33%) expressed the view that fewer categories would suffice.

### 3.3. Category Instability Comparison

Eight (72.7%) of the eleven categories included in the second survey showed decreased instability values after we revised the HL7 categories and definitions (see Table 6). The “Diagnoses” category was highly stable in the first survey, with only one request out of 74 for a change of definition (1.35%), but was the least stable category in the second survey, with a category sum (146.5) accounting for the highest proportion (47.79%) of the total weighted sum of disagreements and partial agreements (306.5). Counter to expectations, the category “Other” appeared more frequently in the second survey than in the first survey, with ten out of twelve participants suggesting that more categories were necessary.

## 4. Discussion

This is the first study to assess physician perspectives on the HL7 Information Sensitivity Policy categories.

### 4.1. Recommendations for the Future Development of HL7 Information Sensitivity Policy Categories

1. **Involve physicians and incorporate their perspectives to ensure that the categories accurately reflect data sensitivity in clinical practice.** In the first survey, physicians recommended significant revisions of the HL7 categories. The recommendations included adding 19 new categories (21.27% suggestions), relocating 7 categories (7.44% suggestions), removing or renaming 4 categories (5.31% suggestions), and revising 25 sensitive data definitions (65.95% suggestions).
2. **Incorporate patient-friendly language and inclusive terminology in the category names and definitions to empower patients to understand and make informed decisions about sharing their sensitive medical records.** From the 94 comments that we received, we found that 22.34% of those included renaming sensitive data categories and definitions to make them more inclusive and patient friendly. Improving the readability and accessibility of the categories can enhance patients’ comprehension and engagement in their own healthcare. Incorporating plain language and considering cultural and linguistic diversity are essential steps toward achieving this goal [19,20]. Researchers and taxonomy developers should collaborate with patient advocacy groups and employ user-centered design approaches to ensure that the taxonomy is patient-centric and empowers individuals to participate in their care actively [21].
3. **Validate and refine the categories using real-world patient data, instead of hypothetical questions, to obtain more insightful outcomes.** During the second survey, disagreements and partial agreements in participants’ data categorizations revealed differences in perspectives on health data. Some participants adopted a broad and context-driven perspective on data categorizations. For example, “Medication Reconciliation (procedure)” was categorized under “Medications”, “Closed fracture of hip” under “Violence” and “Diarrhea symptom (finding)” under “Infectious diseases”. Soni et al.’s study also reported that often, patients with behavioral health conditions adopted a context-based approach to categorize their own health data [1]. For instance, a patient categorized laxatives as “Mental health” information because laxatives were prescribed to address the side effects of their mental health medications.
4. **Assess the stability of categories to guide efforts to prioritize machine-interpretable sensitive data segmentation (e.g., Consent2Share and ONC LEAP-CDS [22]) efforts to enhance integration and interoperability**. During the first survey, more than half of the participants (58.33%) indicated the need for additional categories. Understanding which categories are more stable will help to prioritize efforts to create machine-interpretable code sets to support electronic-based granular data sharing engines [23]. The “Diagnoses” class that was considered the most stable (1.35% out of 74 suggestions for change) in the first survey became the least stable (54.31% out of 232 disagreements) in the second survey. Some of the disagreements between “Diagnoses” and “Other” (e.g., “Alanine aminotransferase [Enzymatic activity/volume] in Serum or Plasma” was categorized as “Diagnoses” and “Other”) suggest the need for adding a new category, “Laboratory/Diagnostic test”. As a participant stated: *“A consideration is to break diagnoses and diagnostic tests into separate categories, since there is a one-to-many relationship”.*

### 4.2. Limitations

It is possible that the study participants’ responses were influenced by the offer of paper co-authorship. To make up for it, the study only revealed a few specifics regarding its goals and design.

This study is a continuation of Grando et al.’s research [10], wherein two physicians were asked to reach agreements in the categorization of 584 unique data health data items. This research increased the sample size to 12 physicians, achieved diversity in physician demographics (age, gender, race, medical specialty and subspecialty, and healthcare organization), and paired physicians to allow for agreements, disagreements, and partial agreements in data item categorizations. On the other hand, we were not able to recruit physicians practicing 11 to 20 years after graduation.

In addition, the lack of an interview follow-up with the participants limits the opportunity to explore their perspectives in greater detail.

### 4.3. Future Work

Future research endeavors should capture a broader range of stakeholder perspectives, including healthcare professionals and patients, to ensure the development of more comprehensive and consensus-based sensitive data categories [1,24,25,26].

The revised categories will be used in a follow-up two-phase interview study involving 24 health providers. Study participants will assess if having access to patients’ EHR influences the way they categorize sensitive data items using the revised categories.

The revised categories will be used in the pilot testing of the ONC Leap Computable Consent Project [22] with the same 26 FHIR synthetic patient medical records that were used for this study.

## 5. Conclusions

The HL7 Information Sensitivity Policy categories have the potential to advance the development of machine-interpretable sensitive data category definitions, enabling patient-driven consent and enhancing sensitive data privacy.

This study provides valuable recommendations for the future development of HL7 Information Sensitivity Policy categories: incorporate physicians’ viewpoints, validate the categories using patient data or/and include patients’ perspectives, and develop patient-centric category specifications.

Future work will further evaluate the HL7 Information Sensitivity Policy categories with physicians using real patient data.

## Figures and Tables

**Table 1:** HL7 Information Sensitive Policy categories.

Proposed Categories for the First Survey	Modified Categories for the Second Survey
**Behavioral health information** Danger to self or others Emotional disturbance information Mental health information Psychiatry disorder information Psychotherapy notes information Substance use disorder information Alcohol use disorder information Opioid use disorder information	**Behavioral health** Danger to self or others Mental health Psychiatry Psychotherapy notes Substance use ▫ Alcohol use ▫ Opioid use
**Demographic information** Date of birth information Gender and sexual orientation information Living arrangement information Marital status information Patient location Race information Religion information	**Demographics**
**Diagnosis information**	**Diagnoses**
**Disabilities** Cognitive disability information Developmental disability	**Disabilities** Cognitive disabilities Developmental disabilities
**Drug information** Pain management	**Medication** Opioid use
**Genetic disease information** Sickle cell disease	**Genetics** Sickle cell disease
**Infectious diseases** HIV/AIDS information Sexually transmitted disease information	**Infectious diseases** HIV/AIDS Sexually transmitted diseases
**Sexual and reproductive health** Pregnancy information	**Sexual and reproductive health** Gender and sexual orientation Pregnancy Sexually transmitted diseases
**Social determinants of health** Living arrangement information Marital status information Patient location	**Social determinants of health** Living arrangements Marital status Nutrition and diet Patient location
**Violence information** Military sexual trauma information Sexual assault, abuse, or domestic violence	**Violence** Domestic violence Military sexual trauma Sexual violence

**Table 2:** Demographics of the study participants (*n* = 12).

Demographics	Freq. (%)
**Age (years)**	
<30	5 (41.66%)
31–40	3 (25.00%)
41–50	1 (8.33%)
51–60	2 (16.66%)
>60	1 (8.33%)
**Gender**	
Male	8 (66.66%)
Female	4 (33.33%)
**Years since graduation from medical school**	
<5	7 (58.33%)
6–10	1 (8.33%)
11–15	0 (0.00%)
16–20	0 (0.00%)
>20	4 (33.33%)
**Medical specialty**	
Internal Medicine	3 (25.00%)
Pediatrics	2 (16.66%)
Emergency Medicine	1 (8.33%)
Family Medicine	1 (8.33%)
General Physician	1 (8.33%)
Obstetrics and Gynecology	1 (8.33%)
Pathology	1 (8.33%)
Preventive Medicine	1 (8.33%)
Psychiatry	1 (8.33%)
Subspecialty	
**No Subspecialty**	9 (66.66%)
Biomedical and Health Informatics	1 (8.33%)
Cancer Research	1 (8.33%)
Hematology-Oncology	1 (8.33%)

**Table 3:** Counts and heat map for total number of suggestions received for each category from initial survey.

Categories	Relocate	Remove/ Rename	Redefine		
Behavioral health	1	3	15	**Count of**	
Demographics	1	0	12	**Suggestions ^a^**	
Diagnoses	0	0	1	Lower	
Disabilities	0	0	10		
Drugs	1	1	2		
Genetic diseases	0	0	3		
Infectious diseases	2	0	3		
Sexual and reproductive health	0	0	2		
Social determinants of health	2	1	6	Higher	
Violence	0	0	8		

^a^ Table cell color corresponds to relative frequency of suggestions across data categories.

**Table 4:** **1:** Survey comments suggesting addition of new categories. **2:** Survey comments suggesting relocation of categories. **3:** Survey comments suggesting removal or renaming of categories. **4:** Survey comments suggesting redefinition of categories.

1			
Category	Freq. of Comments	Participant Quotes (Inclusive and Patient-Friendly Comments are in *Italics*)	Changes Made to Categories
Family medical history	2	*Individuals may not wish to share “Family medical history” and “Health risk factors” with various agents, such as insurance providers, who may discriminate based on this history.* No rationale provided.	No change (Protected information but not sensitive from the patient’s point of view).
Behavioral health lifestyle factors	1	Add a section on “Behavioral health lifestyle factors” to “Behavioral health”.	No change (Included in “Behavioral health” definition).
Birth control history	1	“Birth control history” can be added to “Sexual and reproductive health”.	No change (Included in “Sexual and reproductive health” definition).
Bullying in school	1	No rationale provided.	No change (Included in “Violence” definition).
Childhood adversity	1	No rationale provided.	No change (Included in “Violence” definition).
Genomic information	1	“Genomic information” may contain sensitive information that is not currently known, but may yield sensitive information in the future, and may be different than “Genetic Disease” which is associated with established diagnosis.	No change (“Genetics” is more patient related and “Genomics” is researchers related term).
Gun violence	1	No rationale provided.	No change (Included in “Violence” definition).
Healthy diet	1	Access to a “Healthy diet” (e.g., food desserts) is missing from “SDOH”.	“Nutrition and diet” are added as a subcategory under “Social determinants of health”.
Health risk factors	1	*Individuals may not wish to share “Family medical history and “Health risk factors” with various agents, such as insurance providers, who may discriminate based on this history.*	No change (Protected information but not sensitive from the patient’s point of view).
HIPAA patient identifiers	1	Suggest adding to demographics a category to cover some of the “HIPAA patient identifiers”: https://www.luc.edu/its/aboutits/itspoliciesguidelines/hipaainformation/18hipaaidentifiers/ (accessed on 1 June 2023).	No change (Included in “Demographics” definition).
Human trafficking issues	1	No rationale provided.	No change (Included in “Violence” definition).
Laboratory and diagnostic tests	1	No rationale provided.	No change (Protected information but not sensitive from the patient’s point of view).
Military combat traumas	1	No rationale provided.	1. No change (Included in “Mental health” definition).
Personality disorders	1	Should “Personality disorder” be there too in “Behavioral health”?	No change (Included in “Behavioral health” definition).
Place of employment and internet	1	No rationale provided.	No change (Included in “Demographics” definition).
Psychiatry	1	“Psychiatry” and “Substance abuse drugs” are missing from this category.	“Psychiatry” added as a subcategory under “Behavioral health”.
Physical impairment	1	*The category of disabilities should include “Physical impairments”.*	No change (Included in “Disabilities” definition).
Social situations	1	No rationale provided.	No change (Included in “Social determinants of health” definition).
Substance abuse drugs	1	“Psychiatry” and “Substance abuse drugs” are missing in “Behavioral health”.	No change (Included in “Substance use” definition).
**2**			
**Category**	**Freq. of comments**	**Quote (Inclusive and patient-friendly comments are in *italics*)**	**Changes made to Categories**
Drugs	1	“Drugs” might be included under “Diagnosis”.	No change.
Gender and sexual orientation	1	Should “Gender and sexual orientation” be under “Sexual and reproductive health”?	“Gender and sexual orientation” added as a subcategory under “Sexual and reproductive health”.
HIV/AIDS	1	“HIV/AIDS” should be considered inside the subgroup of “Sexually transmitted diseases”.	No change.
Living arrangements	1	Include in “Demographics”.	No change.
Marital status	1	Include in “Demographics”.	No change.
Psychiatric disorders	1	“Psychiatric disorders” can be the overarching heading for these instead of “Behavioral health”.	No change.
Sexually transmitted diseases	1	I would include sexually transmitted diseases under “Sexual and reproductive health”.	“Sexually transmitted diseases” added as a subcategory under “Sexual and reproductive health”.
**3**			
**Category**	**Freq. of comments**	**Quote (Inclusive and patient-friendly comments are in *italics*)**	**Changes made to Categories**
Emotional disturbance	2	*Should this be labeled “Mood disorder”?* No rationale provided.	Category removed.
Behavioral health	1	Perhaps “Psychosomatic symptoms” would be a better term than stress-related physical symptoms which could also refer to physical stress from occupational work or athletic training.	No change.
Drugs	1	*Rename as “Substances”.*	Renamed “Drugs” as “Medications”.
Social determinants of health	1	“Demographics” should have a category like “Other SDOH” instead of having “SDOH” a separate category.	No change.
**4**			
**Category**	**Freq. of comments**	**Quote (Inclusive and patient-friendly comments are in *italics*)**	**Changes made to categories**
Sexual assault, abuse or domestic abuse	5	*Missing nonconsensual and the outcome of this violence such as injury, mental health disorder, or PTSD. I agree women experience assault more. But the way it’s written makes it seem like men do not experience assault.* *Elder abuse?* No rationale provided. No rationale provided. No rationale provided.	Definition changed.
Disabilities	4	Disability” results from the interaction between individuals with a health condition (such as cerebral palsy, Down syndrome and depression) and personal and environmental factors which affect their quality of life including but not limited to negative attitudes, inaccessible transportation and public buildings, and limited social support. 2. “Disability” is a physical, mental, sensory, etc… impairment that limits one or major life activities of an individual. Disabilities can be congenital or acquired; can also be temporary or long-term/permanent. 3. Include conditions like Low IQ, amputation, Down’s, etc. 4. No rationale provided.	Definition changed.
Race	4	*If this is going to be used in a medical context, I would remove “hierarchical” and “marginalize”. Although the historic context is important, in medicine, “race” is sometimes important to identify population-specific variations (e.g., hypertension treatment varies).* *“Race”? self-identified or any component of familial ancestry which may have cultural and biologic implications.* “Race” is a part of demographics. “Race” as a social construct is a recent theory/scientific trend that is far from being settled and is charged with politics. Like “gender and sexual orientation”, the theory that “race” is a social construct is controversial and shouldn’t be taken as a fact.	Category removed.
Cognitive disability	3	This definition provides examples of conditions, however, it should include aspects of cognitive impairment such as “…and other dementias which result in difficulties with mental processes such as learning, concentrating, memory, decision making,”. Include what “cognitive disability” means: thinking, learning, problem solving, communication, memory, etc. There are also learning disabilities. Will get push back on for instance autism spectrum, is it a “cognitive disability”? otherwise the rest of examples seem appropriate. Sometimes “neurocognitive” can be tested for. What is Neurodiversity?	No change.
Danger to self or others	3	Change the first sentence to “…A person may be dangerous to self and/or others when he or she has recently threatened others, has suicidal thoughts, attempt suicide, or threatened or attempted serious bodily injury to self or others. Infections (especially sexually transmitted diseases, among others) could also be considered in this situation. That is privileged information that can be shared if someone is in “danger” of contracting the disease/infection. *A person with dementia may likewise be unable to care for themselves and there are other social determinants that may add to difficulty, not simply a psychiatric condition. DTS can also be very passive and not verbalize.*	Definition changed.
Developmental disability	3	Add this aspect from the CDC Facts definition listed, “which begins in the developmental phase of a person’s lifetime and can be permanent. *Concern for use “disability” trying to move away, the new term is neurodiversity.* I consider that in this definition should emphasize the establishment of the condition during the developmental stages of growth or secondary to conditions established within the frame of milestones development.	Definition changed.
Gender and sexual orientation	3	I would reword “blend of both”. The concept of sex being different than gender identity and sexual orientation is new and was derived from flawed experiments and sexual abuse (see John Money). Even if western medical trends are moving to accept those theories as true, it is far from “evidence-based” and always has political undertones. It should not be taken lightly or politically. *I think this statement only defines gender, but not defines sexual orientation that is also included in the subtitle. I consider that sexual orientation should be defined as one’s sexual attraction to others sex. Heterosexual if the sexual attraction is directed towards people of the opposite sex, homosexual if is directed to people from the same sex and bisexual if attracted to both sexes.*	Definition changed.
Genetic disease	3	Perhaps add that genetic diseases may be inherited or acquired, 2nd paragraph of this definition from NIH: https://www.genome.gov/For-Patients-and-Families/Genetic-Disordersmust it be demonstrated by genomic testing? (accessed on 1 June 2023). I think it’s worth mentioning that some genetic diseases are hereditary or runs in the family line. No rationale provided.	Definition changed.
Infectious disease	3	Such as using microorganisms instead of germ. Also, suggest adding “host response” from this reference here: https://www.ncbi.nlm.nih.gov/books/NBK800 (accessed on 1 June 2023). Change germs to “pathogenic microorganisms”. Also, mention that they “enter the body and multiply, leading to harmful effects on the person/host”. Diseases are transmissible. It might be worth mentioning that these are diseases whose transmission can be minimized or prevented through limitation of contact (direct or indirect) and through strict hygiene and distancing.	Definition changed.
Living arrangements	3	*I believe households are too descriptive, what if a patient is homeless?* *Is there a provision for “homeless”, variously referred to as “unhoused”, “staying on a friend’s couch”, in a car, etc.* No rationale provided.	Definition changed.
Marital status	3	Add common law marriage which is allowed in a minority of states. *Question, though “never married”, what about “domestic partners”/significant others in a “stable or long-term relationship?* One thing that should be included is if there is a planned divorce or separation which is a major stressor.	Definition changed.
Opioid use disorder	3	*Suggest adding social well-being or “significant impact on quality of life”. Also add “negative affects the person’s personal/work performance”.* I would change “Illness” to disease. No rationale provided.	Definition changed.
Psychiatric disorder	3	It affects the patient’s thoughts, emotions, behavior and overall functioning. It often results from a combination of genetic, biological, psychological and environmental factors. It is not defined solely by the presence of specific symptoms but by the impact of these symptoms in the patient’s life. *Concerned about “…not readily controlled by individuals…” My concern is that this definition makes a psychiatric disorder seem volitional which when I have observed a person having acute psychotic break, the individual may not be self-aware of what is occurring.* “Psychiatry disorders” should also include an historic view and the understanding that psychiatry as we know it is relatively new and has had many critics and detractors like Foucault.	Definition changed.
Social determinants of health	3	Suggest framing at the 5 domains from this resource from HHS: https://health.gov/healthypeople/priority-areas/social-determinants-health (accessed on 1 June 2023). How would you qualify “food as medicine”. A diabetic in a food desert and poor access to nutrition, would you still call that non-medical? Caveat: this definition exposes a theory. This is not a scientific fact without controversies.	Definition changed.
Substance use disorder	3	*This is a definition of diagnostic criteria instead of a definition of the disorder. I would also include something on how substance dependence interferes with the patient’s daily tasks and functions.* Definition works however, why separate ETOH and Opioid? What about methamphetamine, etc.? No rationale provided.	Definition changed.
Drugs	2	Incorporate the definition of drugs from the NCI here: https://www.cancer.gov/publications/dictionaries/cancer-terms/def/drug (accessed on 1 June 2023). How specific? classes of drugs, drugs of abuse, therapeutic/prescribed or self-medicating?	Definition changed.
Psychotherapy notes	2	Would reword “contents of conversation during a private counseling session”. Also, could also be by a psychiatrist (not only psychologists). Finally, “can contain information that is sensitive to include in medical records”. “Psychotherapy notes” have to be identified as such and filed SEPARATELY from the rest of the person’s medical records otherwise they are not deemed to be part of the patient’s regular medical record.	Definition changed.
Violence	2	*What if the person is a perpetrator, not just a victim? Convicted?* Should include the issue of law enforcement involvement.	No change.
Diagnosis	1	“Diagnosis” is the result of the process of identifying a disease, condition, or injury by obtaining a health history including signs and symptoms, performing a physical exam, and performing tests (such as blood tests, imaging tests, and biopsies).	No change.
Mental health	1	Suggest adding to the descriptors of well-being “psychological, emotional, and social”.	Definition changed.
Military sexual trauma	1	This should include physical trauma in addition to psychological trauma resulted from a sexual assault.	No change.
Patient location	1	*What if there is not an address?*	Category removed.
Pregnancy	1	This should include issues with birth control.	No change.
Religion	1	*Institutionalized or personal? atheism? how to account for Native American “religion”?*	Category removed.
Sexually transmitted diseases	1	Indirect transmission is also possible (e.g., needles).	No change.

**Table 5:** Counts and heat map for within-pair agreements, partial agreements, and disagreements for each category.

Category	Agree	Partially Agree	Disagree		
Behavioral health	2	14	6	**Count**	
Demographics	8	8	12	Lower	
Diagnoses	70	41	126		
Disabilities	2	3	3		
Medications	37	23	38		
Genetics	0	2	2		
Infectious diseases	0	14	10		
Sexual and reproductive health	5	12	2		
Social determinants of health	1	12	9		
Violence	0	1	1		
Other	94	19	23	Higher	

**Table 6:** Heat map of category instability values for the first and second surveys.

	Category Instability			
Categories	First Survey	Second Survey		
Behavioral health	19 (25.67%)	13 (4.24%)	**Instability ^a^**	
Demographics	13 (17.56%)	16 (5.22%)	Lower	
Diagnoses	1 (1.35%)	146.5 (47.79%)		
Disabilities	10 (13.51%)	4.5 (1.46%)		
Drugs	4 (5.40%)	49.5 (16.15%)		
Genetic diseases	3 (4.05%)	3 (0.97%)		
Infectious diseases	5 (6.75%)	17 (5.50%)		
Sexual and reproductive health	2 (2.70%)	8 (2.60%)		
Social determinants of health	9 (12.16%)	15 (4.89%)		
Violence	8 (10.81%)	1.5 (0.48%)		
Other	0 (0.00%)	32.5 (10.60%)	Higher	

^a^ Table cell color corresponds to relative magnitude of category instability across surveys.

## Data Availability

The data presented in this study are available in Supplementary S1.

## Supplementary Materials

The following supporting information can be downloaded at: https://www.mdpi.com/article/10.3390/healthcare11212845/s1, Supplementary S1: Data set; contain 600 health data items that were used to validate physicians’ perspectives on revised categories. Supplementary S2: Information Sensitive Policy Value Set Categories; contain proposed sensitive data categories. Supplementary S3: Second survey results; contain health data items categorization by six pairs of physicians as agreement, partial agreement, or disagreement. Supplementary S4: Revised Information Sensitive Policy Value Set Categories; contain revised sensitive data categories.
