# Supplementary material for: Physicians’ Perspectives on HL7 Information Policy Sensitive Value Set: A Validation Study through Health Concept Categorization
Source: Healthcare (Basel). 2023 Oct 28;11(21):2845. doi: 10.3390/healthcare11212845 (PMC10647660; doi:10.3390/healthcare11212845)
Supplement: Supplementary file 1 [file healthcare-11-02845-s001.zip › Supplementary S2.pdf]

<u>Proposed Information Sensitivity Policy Value Set Categories</u>

NOTE: Categories comes from the HL7 Information Sensitivity Policy Value Set, except for *the italic* categories from Banerjee et al., study.

1. Behavioral health information

a. Mental health information

b. Substance use disorder information

i. Alcohol use disorder information

ii. Opioid use disorder information

c. Psychiatry disorder information

d. Psychotherapy notes information

e. *Danger to Self or Others*

f. Emotional disturbance information

2. Demographic information

a. Date of birth information

b. Gender and sexual orientation information

c. Living arrangement information

d. Marital status information

e. Patient location

f. Race information

g. Religion information

3. Diagnosis information

4. ***Disabilities***

a. Cognitive disability information

b. Developmental disability

5. Drug information

a. Pain Management

6. Genetic disease information

a. Sickle cell disease

7. ***Infectious diseases***

a. Sexually transmitted disease information

b. HIV/AIDS information

8. Sexual and Reproductive health

a. Pregnancy information

9. ***Social determinants of health***

a. Patient location

b. Living arrangement information

c. Marital status information

10. Violence information

a. Sexual assault, abuse, or domestic violence

b. Military sexual trauma information

Value Set Definitions

1. Behavioral health information - Behavioral health generally refers to mental health and substance use disorders, life stressors and crises, and stress-related physical symptoms. Behavioral health care refers to the prevention, diagnosis and treatment of those conditions.

https://www.ama-assn.org/delivering-care/public-health/what-behavioral-health

1. a. Mental Health Information - Mental health, as defined by the World Health Organization, is "a state of well-being in which every individual realizes his or her own potential, can cope with the normal stresses of life, can work productively and fruitfully, and is able to make a contribution to his or her community."

https://www.paho.org/en/topics/mental-health#:~:text=The%20World%20Health%20Organization%20(WHO,to%20his%20or%20her%20community%E2%80%9D.

1. b. Substance use disorder information - A substance use disorder (SUD) is a mental disorder that affects a person's brain and behavior, leading to a person's inability to control their use of substances such as legal or illegal drugs, alcohol, or medications. Symptoms can range from moderate to severe, with addiction being the most severe form of SUDs.

https://www.nimh.nih.gov/health/topics/substance-use-and-mental-health#:~:text=A%20substance%20use%20disorder%20(SUD,most%20severe%20form%20of%20SUDs.

1. b. i. Alcohol use disorder information - Alcohol use disorder is a pattern of alcohol use that involves problems controlling your drinking, being preoccupied with alcohol or continuing to use alcohol even when it causes problems. This disorder also involves having to drink more to get the same effect or having withdrawal symptoms when you rapidly decrease or stop drinking. Alcohol use disorder includes a level of drinking that's sometimes called alcoholism.

https://www.mayoclinic.org/diseases-conditions/alcohol-use-disorder/symptoms-causes/syc-20369243

1. b. ii. Opioid use disorder information - Opioid use disorder is a complex illness characterized by compulsive use of opioid drugs even when the person wants to stop, or when using the drugs negatively affects the person's physical and emotional well-being.

https://www.hopkinsmedicine.org/health/conditions-and-diseases/opioid-use-disorder

1. c. Psychiatry disorder information - Psychiatric disorders are behavioral, emotional, or cognitive dysfunctions that are not readily controlled by the individual and are related to clinically significant distress or impairment in one or more areas including social, occupational, and interpersonal functioning. The behavioral and emotional response is beyond a culturally expected response to common life stressors and losses. While many disordered states violate the social norms of a given society, social deviance in itself is not a sufficient criteria for a determination of mental disorder. [Textbook: Encyclopedia of Quality of Life and Well-Being Research, pp 5134–5141].

https://link.springer.com/referenceworkentry/10.1007/978-94-007-0753-5_2303

1. d. Psychotherapy notes information - psychotherapy notes are defined as "notes recorded in any medium by a mental health professional documenting or analyzing the contents of conversation during a private counseling session." These notes, which capture the psychologist's impressions about the patient and can contain information that is inappropriate for a medical record, are similar to what psychologists have historically referred to as "process notes." [American Psychological Association]

https://www.apa.org/monitor/feb03/hipaa

1. e. *Danger to Self or Others-* Danger to self or others means: With respect to other persons, that the individual poses a substantial risk of physical harm to another person or persons, as manifested by evidence of recent homicidal or other violent behavior by the person in question, or by evidence that others are placed in reasonable fear of violent behavior and serious physical harm to them, as evidenced by a recent overt act, attempt, or threat to do serious physical harm by the person in question.

https://www.lawinsider.com/dictionary/danger-to-self-or-others

1. f. Emotional disturbance information - Emotional disturbance means a condition exhibiting one or more of the following characteristics over a long period of time and to a marked degree that adversely affects a child's educational performance:

(A) An inability to learn that cannot be explained by intellectual, sensory, or health factors.
(B) An inability to build or maintain satisfactory interpersonal relationships with peers and teachers.
(C) Inappropriate types of behavior or feelings under normal circumstances.
(D) A general pervasive mood of unhappiness or depression.
(E) A tendency to develop physical symptoms or fears associated with personal or school problems.

https://dphhs.mt.gov/schoolhealth/chronichealth/developmentaldisabilities/emotionaldisturbance

2. Demographic information - Demographic data is information about groups of people according to certain attributes such as age, gender, place of residence, and can include socio-economic factors such as occupation, family status, or income.

https://en.ryte.com/wiki/Demographic_Data

2. a. Date of birth information - A person's date of birth denotes the day, month and year that an individual was born on. This is an essential piece of personal data, that is often used on official ID documents, such as your passport, driver's license, and official birth certificate.

https://www.usbirthcertificates.com/glossary/date-of-birth#:~:text=Date%20of%20Birth%20definition,license%2C%20and%20official%20birth%20certificate.

2. b. Gender and sexual orientation information - One's innermost concept of self as male, female, a blend of both or neither – how individuals perceive themselves and what they call themselves. One's gender identity can be the same or different from their sex assigned at birth.

An inherent or immutable enduring emotional, romantic or sexual attraction to other people. Note: an individual's sexual orientation is independent of their gender identity.

https://www.hrc.org/resources/sexual-orientation-and-gender-identity-terminology-and-definitions

2. c. Living arrangement information - Living arrangements refer to the structure and composition of one's household, including the number of household members and their relationships to each another. Living arrangements include both single-person households (i.e., living alone) and multi-person households, including those comprised of persons with familial ties (e.g., living with a spouse or adult children) or those without familial ties (e.g., living with a partner or others related or unrelated). Living arrangements have also been examined in terms of intergenerational co-residence among older adults and their adult children – households that are often shaped by one's individual needs, family structure, and cultural contexts. [Textbook: Encyclopedia of Gerontology and Population Aging, pp 1–7]

https://link.springer.com/referenceworkentry/10.1007/978-3-319-69892-2_324-1

2. d. Marital status information - Marital status is the legally defined marital state. There are several types of marital status: single, married, widowed, divorced, separated and, in certain

cases, registered partnership. Never married persons are persons who never got married in concordance with valid regulations.

https://ec.europa.eu/eurostat/statistics-explained/index.php?title=Glossary:Marital_status#:~:text=Marital%20status%20is%20the%20legally,in%20concordance%20with%20valid%20regulations.

2. e. Patient location - Patient address means the current geographic location of the patient's residence. If the patient's address is in the care of another person or entity, the address of that person or entity is the "patient address" of the record. When alternate addresses are possible, they must be recorded in the order of preference.

https://www.lawinsider.com/dictionary/patient-address#:~:text=Patient%20address%20means%20the%20current,%22patient%20address%22%20of%20record.

2. f. Race Information - Race is a social construct used to group people. Race was constructed as a hierarchal human-grouping system, generating racial classifications to identify, distinguish and marginalize some groups across nations, regions and the world. Race divides human populations into groups often based on physical appearance, social factors and cultural backgrounds.

https://www.genome.gov/genetics-glossary/Race

2. g. Religion information - Religion is a collection of cultural systems, belief systems, and worldviews that relate humanity to spirituality and, sometimes, to moral values. Many religions have narratives, symbols, traditions and sacred histories that are intended to give meaning to life or to explain the origin of life or the universe.

https://www.coe.int/en/web/compass/religion-and-belief#:~:text=Religion%20is%20a%20collection%20of,of%20life%20or%20the%20universe.

3. Diagnosis information - The process of identifying a disease, condition, or injury from its signs and symptoms. A health history, physical exam, and tests, such as blood tests, imaging tests, and biopsies, may be used to help make a diagnosis.

https://www.cancer.gov/publications/dictionaries/cancer-terms/def/diagnosis

4. *Disabilities* - Disability results from the interaction between individuals with a health condition, such as cerebral palsy, Down syndrome and depression, with personal and

environmental factors including negative attitudes, inaccessible transportation and public buildings, and limited social support.

https://www.who.int/health-topics/disability#tab=tab_1

4. a. Cognitive disability information - Cognitive disabilities is a term that refers to a broad range of conditions that include intellectual disability, autism spectrum disorders, severe, persistent mental illness, brain injury, stroke, and Alzheimer's disease and other dementias.

https://www.fcc.gov/cognitive-disabilities#:~:text=%E2%80%9CCognitive%20disabilities%E2%80%9D%20is%20a%20term,Alzheimer%27s%20disease%20and%20other%20dementias.

4. b. Developmental disability - Developmental disabilities are a group of conditions due to an impairment in physical, learning, language, or behavior areas.

https://www.cdc.gov/ncbddd/developmentaldisabilities/index.html

5. Drug information - Drug information refers to "current, critically examined, relevant data about drugs and drug use in a given patient in a particular situation.

https://link.springer.com/chapter/10.1007/978-981-32-9779-1_14

5. a. *Pain Management* - Multidisciplinary pain management (MDPM) refers to an integrated approach in which multimodal treatment is provided by a multidisciplinary team collaborating in assessment and treatment using a shared biopsychosocial model and goals. An example is the prescription of an antidepressant medication by a physician alongside an exercise plan from a physiotherapist and training in pain self- management skills from a psychologist.

https://www.iasp-pain.org/resources/toolkits/pain-management-center/chapter1/

6. Genetic disease information - A genetic disorder is a disease caused in whole or in part by a change in the DNA sequence away from the normal sequence. Genetic disorders can be caused by a mutation in one gene (monogenic disorder), by mutations in multiple genes (multifactorial inheritance disorder), by a combination of gene mutations and environmental factors, or by damage to chromosomes (changes in the number or structure of entire chromosomes, the structures that carry genes).

https://www.genome.gov/For-Patients-and-Families/Genetic-Disorders

6. a. Sickle cell disease - Sickle cell disease (SCD) is a group of inherited red blood cell disorders. Red blood cells contain hemoglobin, a protein that carries oxygen. Healthy red blood cells are round, and they move through small blood vessels to carry oxygen to all parts of the body. In someone who has SCD, the hemoglobin is abnormal, which causes the red blood cells to become hard and sticky and look like a C-shaped farm tool called a "sickle." The sickle cells die early, which causes a constant shortage of red blood cells. Also, when they travel through small blood vessels, they get stuck and clog the blood flow. This can cause pain and other serious complications (health problems) such as infection, acute chest syndrome and stroke.

https://www.cdc.gov/ncbddd/sicklecell/facts.html

7. *Infectious diseases* - Infectious diseases are illnesses caused by germs (such as bacteria, viruses, and fungi) that enter the body, multiply, and can cause an infection.

https://www.cdc.gov/ncezid/who-we-are/index.html#:~:text=Infectious%20diseases%20are%20illnesses%20caused,from%20one%20person%20to%20another.

7. a. Sexually transmitted disease information - Sexually transmitted diseases (STDs) are infections transmitted from an infected person to an uninfected person through sexual contact. STDs can be caused by bacteria, viruses, or parasites. Examples include gonorrhea, genital herpes, human papillomavirus infection, HIV/AIDS, chlamydia, and syphilis.

https://www.niaid.nih.gov/diseases-conditions/sexually-transmitted-diseases

7. b. HIV/AIDS information - Acquired immunodeficiency syndrome (AIDS) is a chronic, potentially life-threatening condition caused by the human immunodeficiency virus (HIV). By damaging your immune system, HIV interferes with your body's ability to fight infection and disease.

https://www.mayoclinic.org/diseases-conditions/hiv-aids/symptoms-causes/syc-20373524

8. Sexual and Reproductive health - Good sexual and reproductive health is a state of complete physical, mental and social well-being in all matters relating to the reproductive system. It implies that people are able to have a satisfying and safe sex life, the capability to reproduce and the freedom to decide if, when, and how often to do so.

https://www.unfpa.org/sexual-reproductive-health

8. a. Pregnancy information- The condition between conception (fertilization of an egg by a sperm) and birth, during which the fertilized egg develops in the uterus. In humans, pregnancy lasts about 288 days.

https://www.cancer.gov/publications/dictionaries/cancer-terms/def/pregnancy

9. ***Social determinants of health*** - Social determinants of health (SDOH) are the nonmedical factors that influence health outcomes. They are the conditions in which people are born, grow, work, live, and age, and the wider set of forces and systems shaping the conditions of daily life. These forces and systems include economic policies and systems, development agendas, social norms, social policies, racism, climate change, and political systems.

https://www.cdc.gov/about/sdoh/index.html

9. a. Patient location - Patient address means the current geographic location of the patient's residence. If the patient address is in the care of another person or entity, the address of that person or entity is the "patient address" of record. When alternate addresses are possible, they must be recorded in the order of preference.

https://www.lawinsider.com/dictionary/patient-address#:~:text=Patient%20address%20means%20the%20current,%22patient%20address%22%20of%20record.

9. b. Living arrangement information - Living arrangements refer to the structure and composition of one's household, including the number of household members and their relationships to each another. Living arrangements include both single-person households (i.e., living alone) and multi-person households, including those comprised of persons with familial ties (e.g., living with a spouse or adult children) or those without familial ties (e.g., living with a partner or others related or unrelated). Living arrangements have also been examined in terms of intergenerational co-residence among older adults and their adult children – households that are often shaped by one's individual needs, family structure, and cultural contexts. [Textbook: Encyclopedia of Gerontology and Population Aging, pp 1–7]

https://link.springer.com/referenceworkentry/10.1007/978-3-319-69892-2_324-1

9. c. Marital status information - Marital status is the legally defined marital state. There are several types of marital status: single, married, widowed, divorced, separated and, in certain cases, registered partnership. Never married persons are persons who never got married in concordance with valid regulations.

https://ec.europa.eu/eurostat/statistics-explained/index.php?title=Glossary:Marital_status#:~:text=Marital%20status%20is%20the%20legally,in%20concordance%20with%20valid%20regulations.

10. Violence information - Violence is the intentional use of physical force or power, threatened or actual, against oneself, another person, or against a group or community, that either results in or has a high likelihood of resulting in injury, death, psychological harm, maldevelopment, or deprivation."

https://www.who.int/groups/violence-prevention-alliance/approach

10. a. Sexual assault, abuse or domestic violence - Domestic and Sexual Violence (DSV) is a collection of behaviors by a perpetrator used to exert power and control over their victim. These behaviors disproportionately effect women across all socio-economic backgrounds.

https://statepatrol.nebraska.gov/services/domestic-and-sexual-violence-program/what-domestic-and-sexual-violence

10. b. Military sexual trauma information - Psychological trauma resulted from a physical assault of a sexual nature, battery of a sexual nature, or sexual harassment that is repeated, unsolicited verbal or physical contact of a sexual nature which is threatening in character and occurred while the veteran was serving on active duty or active duty for training.

https://connect.springerpub.com/content/book/978-0-8261-2779-2/part/part01/chapter/ch01?implicit-login=true
