# Supplementary material for: Physicians’ Perspectives on HL7 Information Policy Sensitive Value Set: A Validation Study through Health Concept Categorization
Source: Healthcare (Basel). 2023 Oct 28;11(21):2845. doi: 10.3390/healthcare11212845 (PMC10647660; doi:10.3390/healthcare11212845)
Supplement: Supplementary file 1 [file healthcare-11-02845-s001.zip › Supplementary S4.pdf]

<u>**Revised Information Sensitivity Policy Value Set Categories**</u>

1. **Behavioral health**
   a. Mental health
   b. Substance use
      i. Alcohol use
      ii. Opioid use
   c. Psychiatry
   d. Psychotherapy notes
   e. Danger to self or others
2. **Demographics**
3. **Diagnoses**
4. **Disabilities**
   a. Cognitive disabilities
   b. Developmental disabilities
5. **Medication**
   a. Opioid use
6. **Genetics**
   a. Sickle cell disease
7. **Infectious diseases**
   a. Sexually transmitted diseases
   b. HIV/AIDS
8. **Sexual and reproductive health**
   a. Pregnancy
   b. Gender and sexual orientation
   c. Sexually transmitted diseases
9. **Social determinants of health**
   a. Patient location

b. Living arrangements

c. Marital status

d. Nutrition and diet

10. Violence

a. Sexual violence

b. Domestic violence

c. Military sexual trauma

Revised Value Set Definitions

1. Behavioral health means the promotion of mental health, resilience and wellbeing; the treatment of mental and substance use disorders; and the support of those who experience and/or are in recovery from these conditions, along with their families and communities.

https://www.samhsa.gov/sites/default/files/samhsa-behavioral-health-integration.pdf

1.a Mental health is a state of well-being in which every individual realizes his or her potential, can cope with the normal stresses of life, can work productively and fruitfully, and can make a contribution to his or her community.

https://www.paho.org/en/topics/mental-health#:~:text=The%20World%20Health%20Organization%20(WHO,to%20his%20or%20her%20community%E2%80%9D.

1.b. Substance use refers to the use of selected substances, including alcohol, tobacco products, drugs, inhalants, and other substances that can be consumed, inhaled, injected, or otherwise absorbed into the body with possible dependence and other detrimental effects

https://www.cdc.gov/nchs/hus/sources-definitions/substance-use.htm

1.b.i. Alcohol use – Alcohol is a psychoactive substance with dependence-producing properties that has been widely used in many cultures for centuries. The harmful use of alcohol causes a high burden of disease and has significant social and economic consequences.

Alcohol (who.int)

1.b.ii. Opioid use – Opioids, also called opiates, are a class of drug. The class includes drugs derived from the opium poppy, such as morphine and codeine. Many opioids are used to treat pain. Some opioids, such as oxycodone, codeine, and morphine, are prescription pain medications. Using these medications for recreation or in a way not prescribed by a doctor can be considered abuse. Other opioids, like heroin, are illegal. Opioids are highly addictive. Abuse can lead to addiction. Both abuse and addiction can cause serious health problems and can lead to death.

Opioids and Related Disorders | Definition and Patient Education (healthline.com)

1.c. Psychiatry is the practice of diagnosing and treating behavioral and mental health disorders. This includes creating personalized treatment plans around an individuals' disorder that can include medication, therapy, counseling, CBT, alternative medicines or any combination of these. Psychiatry is practiced by specially trained doctors or nurse practitioners depending on state law.

https://arraybc.com/defining-behavioral-health

1.d. Psychotherapy notes are recorded in any medium by a mental health professional documenting or analyzing the contents of conversation during a private counseling session. These notes, which capture the psychologist's impressions about the patient and can contain information inappropriate for a medical record, are similar to what psychologists have historically referred to as "process notes."

https://www.apa.org/monitor/feb03/hipaa

1.e. Danger to self or others- A person may be dangerous to self and others when he or she have recently threatened or attempted suicide or some serious bodily injury. He or she may have demonstrated danger of substantial and imminent harm to himself and/or others through some recent act, attempt or threat of the same. 'Dangerous to self' may also include a situation where a person is unable to cater to his nourishment, shelter or self protection without supervision or assistance of another person. Without such supervision and adequate treatment, it is probable that the mentally ill individual may succumb to death, substantial bodily injury or serious physical debilitation or disease.
https://www.lawinsider.com/dictionary/danger-to-self-or-others

2. Demographics includes a patient's name, gender, address, date of birth, social security number, and other personally identifiable information, but shall not include any information regarding a patient's health or medical treatment or the names of any Data Providers that maintain medical records about such patient.

https://www.lawinsider.com/dictionary/demographic-information

3. Diagnoses are the result of the process of identifying a disease, condition, or injury from its signs and symptoms. A health history, physical exam, and tests, such as blood tests, imaging tests, and biopsies, may be used to help make a diagnosis.

https://www.cancer.gov/publications/dictionaries/cancer-terms/def/diagnosis

4. Disabilities result from the interaction between individuals with a health condition, such as cerebral palsy, Down syndrome and depression, with personal and environmental factors including negative attitudes, inaccessible transportation and public buildings, and limited social support.

https://www.who.int/health-topics/disability#tab=tab_1

4.a. Cognitive disabilities refers to a broad range of conditions that include intellectual disability, autism spectrum disorders, severe, persistent mental illness, brain injury, stroke, and Alzheimer's disease and other dementias.

Cognitive Disabilities | Federal Communications Commission (fcc.gov)

4.b. Developmental disabilities are conditions due to an impairment in physical, learning, language, or behavioral areas.

https://www.cdc.gov/ncbddd/developmentaldisabilities/index.html

5. Medication are substances (other than food) used to prevent, diagnose, treat, or relieve symptoms of a disease or abnormal condition. Drugs can also affect how the brain and the rest of the body work and cause changes in mood, awareness, thoughts, feelings, or behavior. Some types of drugs, such as opioids, may be abused or lead to addiction.

https://www.cancer.gov/publications/dictionaries/cancer-terms/def/drug

5.b. Opioid use– Opioids, also called opiates, are a class of drug. The class includes drugs derived from the opium poppy, such as morphine and codeine. Many opioids are used to treat pain. Some opioids, such as oxycodone, codeine, and morphine, are prescription pain medications. Using these medications for recreation or in a way not prescribed by a doctor can be considered abuse. Other opioids, like heroin, are illegal. Opioids are highly addictive. Abuse

can lead to addiction. Both abuse and addiction can cause serious health problems and can lead to death.

Opioids and Related Disorders | Definition and Patient Education (healthline.com)

6. Genetics: Genetics is the scientific study of genes and heredity of how certain qualities or traits are passed from parents to offspring as a result of changes in DNA sequence. A gene is a segment of DNA that contains instructions for building one or more molecules that help the body work. Genetic variations can influence how people respond to certain medicines or a person's likelihood of developing a disease.

Genetics (nih.gov)

6.a. Sickle cell disease is a group of inherited red blood cell disorders. Red blood cells contain hemoglobin, a protein that carries oxygen. Healthy red blood cells are round and move through small blood vessels to carry oxygen to all body parts. In someone with sickle cell disease, the hemoglobin is abnormal, which causes the red blood cells to become hard and sticky and look like a C-shaped farm tool called a "sickle." The sickle cells die early, which causes a constant shortage of red blood cells. Also, when they travel through small blood vessels, they get stuck and clog the blood flow. This can cause pain and other serious complications (health problems) such as infection, acute chest syndrome and stroke.

https://www.cdc.gov/ncbddd/sicklecell/facts.html

7. Infectious diseases are illnesses caused by harmful organisms (pathogens) that get into your body from the outside. Pathogens that cause infectious diseases are viruses, bacteria, fungi, parasites and, rarely, prions. You can get infectious diseases from other people, bug bites and contaminated food, water or soil.

https://my.clevelandclinic.org/health/diseases/17724-infectious-diseases

7.a. Sexually transmitted diseases are infections transmitted from an infected person to an uninfected person through sexual contact. These diseases can be caused by bacteria, viruses, or parasites. Examples include gonorrhea, genital herpes, human papillomavirus infection, HIV/AIDS, chlamydia, and syphilis.

https://www.niaid.nih.gov/diseases-conditions/sexually-transmitted-diseases

7.b. HIV/AIDS - Acquired immunodeficiency syndrome (AIDS) is a chronic, potentially life-threatening condition caused by the human immunodeficiency virus (HIV). By damaging the immune system, HIV interferes with the body's ability to fight infection and disease.

https://www.mayoclinic.org/diseases-conditions/hiv-aids/symptoms-causes/syc-20373524

8. Sexual and reproductive health - Good sexual and reproductive health is a state of complete physical, mental and social well-being in all matters relating to the reproductive system. It implies that people are able to have a satisfying and safe sex life, the capability to reproduce and the freedom to decide if, when, and how often to do so.

https://www.unfpa.org/sexual-reproductive-health

8.a. Pregnancy is the condition between conception (fertilization of an egg by a sperm) and birth, during which the fertilized egg develops in the uterus. In humans, pregnancy lasts about 288 days.

https://www.cancer.gov/publications/dictionaries/cancer-terms/def/pregnancy

8.b. Gender and sexual orientation is one's innermost concept of self as male, female, a blend of both or neither. It is how individuals perceive themselves and what they call themselves. One's gender identity can be the same or different from their sex assigned at birth. Sexual orientation is a part of individual identity that includes "a person's sexual and emotional attraction to another person and the behavior and/or social affiliation that may result from this attraction"

https://www.hrc.org/resources/sexual-orientation-and-gender-identity-terminology-and-definitions

Sexual orientation (apa.org)

8.c. Sexually transmitted diseases are infections transmitted from an infected person to an uninfected person through sexual contact. These diseases can be caused by bacteria, viruses, or parasites. Examples include gonorrhea, genital herpes, human papilloma virus infection, HIV/AIDS, chlamydia, and syphilis.

https://www.niaid.nih.gov/diseases-conditions/sexually-transmitted-diseases

9. Social determinants of health (SDOH) are the conditions in the environments where people are born, live, learn, work, play, worship, and age. Social determinants of health affect a wide range of health, functioning, and quality-of-life outcomes and risks.

https://health.gov/healthypeople/priority-areas/social-determinants-health

9.a. Patient location means the current geographic location of the patient's residence.

https://www.lawinsider.com/dictionary/patient-address#:~:text=Patient%20address%20means%20the%20current,%22patient%20address%22%20of%20record.

9.b. Living arrangements are where a person lives, whether alone or with someone else, is homeless or transient, or lives in an institution (such as a nursing home). Living arrangements also depend on who pays for your food and shelter.

https://www.ssa.gov/ssi/text-living-ussi.htm#:~:text=Your%20living%20arrangement%20is%20where,for%20your%20food%20and%20shelter.

9.c. Marital status is the legally defined marital state. There are several types of marital status: single, married, widowed, divorced, separated and, in certain cases, registered partnership. Never married persons are persons who never got married in concordance with valid regulations.

https://ec.europa.eu/eurostat/statistics-explained/index.php?title=Glossary:Marital_status#:~:text=Marital%20status%20is%20the%20legally,in%20concordance%20with%20valid%20regulations.

9.d. Nutrition and diet: Diet refers to the total amount of food consumed by individuals; whereas nutrition is the process of utilizing food for growth, metabolism and repair of tissues. The relationship between diet and nutrition and health is 2-way; health status can be affected by nutrient deficiency and vice versa.

Chapter 1: Nutrition and Diet - PubMed (nih.gov)

10. Violence is the intentional use of physical force or power, threatened or actual, against oneself, another person, or a group or community, that either result in or have a high likelihood of resulting in injury, death, psychological harm, maldevelopment, or deprivation.

https://www.who.int/groups/violence-prevention-alliance/approach

10.a. Sexual violence - Any sexual act, attempt to obtain a sexual act, unwanted sexual comments or advances, or acts to traffic or otherwise directed against a person's sexuality using

coercion, by any person regardless of their relationship to the victim, in any setting, including but not limited to home and work.

WHO_RHR_12.37_eng.pdf

10.b. Domestic violence is a pattern of abusive behavior in any relationship that is used by one partner to gain or maintain power and control over another intimate partner. Domestic violence can be physical, sexual, emotional, economic, psychological, or technological actions or threats of actions or other patterns of coercive behavior that influence another person within an intimate partner relationship. This includes any behaviors that intimidate, manipulate, humiliate, isolate, frighten, terrorize, coerce, threaten, blame, hurt, injure, or wound someone.

https://www.justice.gov/ovw/domestic-violence

10.c. Military sexual trauma is a psychological trauma resulted from a physical assault of a sexual nature, battery of a sexual nature, or sexual harassment that is repeated, unsolicited verbal or physical contact of a sexual nature which is threatening in character and occurred while the veteran was serving on active duty or active duty for training.
https://connect.springerpub.com/content/book/978-0-8261-2779-2/part/part01/chapter/ch01?implicit-login=true
